# 

**Published:** 1882-06

**Authors:** 


### CONTENTS:

PAGE.

ORIGINAL COMMUNICATIONS:

I. The Differential Diagnosis of the Cause of Sudden Unconsciousness. By R. O. Beard, M.D. .... 561

II. Bemis v. Hayes. By M. P. Hatfield..... 574

SOCIETY REPORTS:

III. American Medical Association. Thirty-third Annual Meeting .... 578

IV. Illinois State Medical Society—A Contribution to the Report of the Committee on Medical Legislation. By E. Ingals, M.D. .... 607

FOREIGN CORRESPONDENCE:

V. Vienna Letter .... 613

DOMESTIC CORRESPONDENCE:

VI. New York Letter .... 618

PAGE.

REVIEWS AND BOOK NOTICES .... 620

Books and Pamphlets Received .... 624

EDITORIAL .... 627

ORIGINAL TRANSLATIONS .... 628

SELECTIONS:

Infant Feeding and Infant Foods .... 634

Prognosis and Treatment of Ankle-joint Disease .... 645

Pemphigus .... 660

ITEMS .... 577, 612, 617, 619, 625, 669

ANNOUNCEMENTS FOR THE MONTH .... 672

NOTICE TO CORRESPONDENTS .... *advts.*

